# Diverse Horizontally-Acquired Gene Clusters Confer Sucrose Utilization to Different Lineages of the Marine Pathogen *Photobacterium damselae* subsp. *damselae*

**DOI:** 10.3390/genes11111244

**Published:** 2020-10-22

**Authors:** Saqr Abushattal, Ana Vences, Alba V. Barca, Carlos R. Osorio

**Affiliations:** Departamento de Microbioloxía e Parasitoloxía, Instituto de Acuicultura, Universidade de Santiago de Compostela, 15782 Santiago de Compostela, Spain; saqr.sulieman@usc.es (S.A.); ana.vences@usc.es (A.V.); albavazquez.barca@usc.es (A.V.B.)

**Keywords:** sucrose, *Photobacterium damselae*, *Vibrio*, *Vibrio damsela*, TCBS, PTS, horizontal gene transfer

## Abstract

The ability to metabolize sucrose is a variable trait within the family *Vibrionaceae*. The marine bacterium *Photobacterium damselae* subsp. *damselae* (*Pdd*), pathogenic for marine animals and humans, is generally described as negative for sucrose utilization (Scr^−^). Previous studies have reported sucrose-utilizing isolates (Scr^+^), but the genetic basis of this variable phenotype remains uncharacterized. Here, we carried out the genome sequencing of five Scr^+^ and two Scr^−^ *Pdd* isolates and conducted a comparative genomics analysis with sixteen additional *Pdd* genomes sequenced in previous studies. We identified two different versions of a four-gene cluster (*scr* cluster) exclusive of Scr^+^ isolates encoding a PTS system sucrose-specific IIBC component (*scrA*), a fructokinase (*scrK*), a sucrose-6-phosphate hydrolase (*scrB*), and a sucrose operon repressor (*scrR*). A *scrA* deletion mutant did not ferment sucrose and was impaired for growth with sucrose as carbon source. Comparative genomics analyses suggested that *scr* clusters were acquired by horizontal transfer by different lineages of *Pdd* and were inserted into a recombination hot-spot in the *Pdd* genome. The incongruence of phylogenies based on housekeeping genes and on *scr* genes revealed that phylogenetically diverse gene clusters for sucrose utilization have undergone extensive horizontal transfer among species of *Vibrio* and *Photobacterium*.

## 1. Introduction

The family *Vibrionaceae* includes a diverse and large number of bacterial species that are ubiquitous in marine habitats. Some species are recognized as pathogens for marine animals and for humans [1,2]. Also, their ecological importance in aquatic ecosystems and their high genetic plasticity have fuelled a major research effort on *Vibrio*, *Photobacterium,* and other genera of this family [3,4,5,6,7]. Most species exhibit a rapid growth in culture and use a large variety of nutrient sources, and the considerable inter- and intraspecies variability in metabolic and nutritional patterns is explained in part by horizontal transfer of gene functions [8,9,10,11]. Historically, carbon source utilization patterns have been extensively used to differentiate species within this family, and this knowledge has inspired the development of selective and differential culture media to enrich for specific taxa and for fast discrimination of some species. The thiosulfate citrate bile-salt sucrose (TCBS) agar, is a classical medium originally developed for the isolation of *Vibrio parahaemolyticus* [12] and is suitable for the isolation of many *Vibrio* and *Photobacterium* species. In addition to compounds that provide selective properties, TCBS contains 2% sucrose and pH indicators, allowing the differentiation between sucrose fermenters that produce yellow colonies (Scr^+^ phenotype) and non-fermenters that produce green colonies (Scr^−^ phenotype). The sucrose fermentation pattern on TCBS is a widespread taxonomic tool for species of the family *Vibrionaceae* [1]. However, very much as a contradiction, the genetic basis of sucrose utilization in species of this family has received scarce attention. Pioneering studies reported the cloning and characterization of sucrose utilization genes in *Vibrio alginolyticus* [13,14,15,16], and later studies reported some aspects on genetics of sucrose utilization in *Vibrio cholerae* [17,18,19,20], whereas no studies have been conducted in species of the genus *Photobacterium* to date.

Understanding the genetic basis for sucrose utilization in different species of the *Vibrionaceae* is essential to elucidate the evolutionary history of the acquisition of this metabolic capability. Sucrose utilization ability exhibits interspecies and intraspecies variability within this family which suggests that sucrose utilization genes are prone to be acquired by horizontal gene transfer. However, the phylogenetic relationships of genes for sucrose metabolism among *Photobacterium* and *Vibrio* species has not been analyzed so far. In addition, much remains to be learned about the role of sucrose utilization genes in the ecology of Vibrios. Sucrose, a disaccharide of glucose and fructose, is one of the most abundant sugars in terrestrial environments and a carbon source used by many bacterial taxa [21]. However, the availability of sucrose in marine environments has received little attention, and of what use is sucrose utilization in the marine environment for bacteria is a question that remains largely unanswered.

The marine pathogen *Photobacterium damselae* subsp. *damselae* (hereafter *Pdd*), formerly known as *Vibrio damsela*, is a member of the family *Vibrionaceae* that thrives in marine and estuarine environments and is a generalist pathogen that causes high financial losses in cultures of marine fish and crustaceans [22]. Moreover, it can cause opportunistic infections in humans that may evolve into a fatal outcome [23]. *Pdd* is considered a genetically diverse subspecies, and horizontal gene transfer largely contributes to the generation of such diversity [22,24,25,26]. *Pdd* typically forms green colonies on TCBS medium, but early studies pointed out that 5% of the isolates produced yellow colonies on TCBS [27]. Since then, a number of studies have described *Pdd* strains that produce yellow colonies on TCBS [24,28,29,30,31], but the genetic basis of such phenotypic variability remains uncharacterized.

In the present study, we accomplished the de novo genome sequencing of 7 *Pdd* isolates, 5 that tested positive for sucrose utilization, and 2 strains that were negative for this trait. Retrieval of additional 16 *Pdd* genome sequences from GenBank database allowed us to conduct an extensive comparative genomics analysis of Scr^+^ and Scr^−^ isolates that unveiled the presence of two distinct genetic variants of a four-gene cluster (*scr* cluster) that is necessary for sucrose utilization as a carbon source in *Pdd*. Notably, the majority of the *scr* clusters appear to be inserted into a hot-spot for recombination of variable DNA sequences within the *Pdd* genome. Based on the phylogenetic analyses of the *scr* clusters of *Pdd* and other species of the family *Vibrionaceae*, and on the reconstruction of species phylogenies by using the sequences of eight conserved housekeeping genes, we propose that sucrose utilization in *Pdd* arose from the horizontal acquisition by different *Pdd* lineages, of at least two distinct versions of gene clusters from species of the *Vibrio* and *Photobacterium* genera. It is also proposed that the exchange of *scr* clusters among species of the family *Vibrionaceae* has been extensive.

## 2. Materials and Methods

### 2.1. Bacterial Strains, Plasmids, and Culture Conditions

*Pdd* strains used here are described in Table 1. Genetically-modified *Pdd* strains, *Escherichia coli* strains, and plasmids used and constructed in this study are described in Table 2. *Pdd* cells were routinely grown at 25 °C on tryptic soy agar and broth supplemented with 1% NaCl (TSA-1 and TSB-1, respectively), supplemented with antibiotics when appropriate. *E. coli* strains were routinely grown at 37 °C in LB broth and LB agar supplemented with antibiotics when appropriate. Antibiotics were used at the following final concentrations: Kanamycin (Km) at 50 µg mL^−1^, ampicillin at 50 µg mL^−1^, and gentamicin at 15 µg mL^−1^. For sucrose fermentation phenotypical tests, *Pdd* strains were grown on TSA-1 plates overnight at 25 °C, and isolated colonies were seeded on thiosulfate-citrate-bile salts-sucrose agar plates (TCBS) and incubated at 25 °C for 18–24 h. Alternatively, API-20E galleries (Biomérieux, Marcy l’Etoile, France) were used for sucrose fermentation assays, following manufacturer’s recommendations.

### 2.2. PCR Assays

All primers used in this study are described in Table 3. For PCR template preparation, isolated single colonies of each strain were picked with sterile tips, resuspended in 20 µL sterile water, and 1 µL suspension was added as template to the PCR tube containing the NZYTaq II 2× green master mix (NZYTech, Lisbon, Portugal), followed by a cell lysis and denaturation step of 5 min at 95 °C. PCR conditions were standardized as follows: 94 °C denaturation for 30 s, primer annealing at 56 °C for 30 s, elongation at 72 °C for 30 s per kilobase. After 30 cycles of amplification, a final elongation step at 72 °C for 1 min was added.

### 2.3. Genome Sequencing

The draft genome sequences of 5 Scr^+^ (DK32, TW250/03, 162bp-OG4A, 189bp-OG7B and 125dy-OG11) and of 2 Scr^−^ *Pdd* strains (CDC-1421-81 and 82dy-OG8) were determined in the present study. High-purity genomic DNA was extracted using the G NOME DNA Kit (MPBio, Irvine, CA, USA). For sequencing libraries preparation, purified DNA was mechanically sheared using an ultrasonicator (Covaris Ltd., Brighton, UK), ends were enzymatically repaired and adaptors (Illumina, San Diego, C, USA) were ligated. Libraries were sequenced using Illumina MiSeq platform (2 × 150-bp paired-end reads). The reads were assembled with SPAdes 3.6 [45]. Genomes were annotated using the NCBI Prokaryotic Genome Annotation Pipeline [46].

### 2.4. Construction of a scrA Mutant in Pdd DK32

A nonpolar deletion of the *scrA* gene was constructed in the Scr^+^ *Pdd* parental strain DK32 using PCR amplification of the ca. 2000 bp sequences upstream and downstream the *scrA* coding sequence, which, when fused together, would result in an in-frame deletion of more than 90% of the coding sequence. Amplification was carried out with primer pair scrA 1-2 to get the 5′ fragment, and with primer pair scrA 3-4 to get the 3′ fragment (Table 3). The PCR products were cut with suitable restriction enzymes and sequentially ligated into the multiple cloning site of pKWS30 plasmid. This cloned fragment involving the deleted region was excised with *Not*I and *Apa*I and then ligated into the Km^r^ suicide vector pNidKan containing the *sacB* gene, which confers sucrose sensitivity, and R6K *ori*, which requires the *pir* gene product for replication. The pNidKan plasmid construct containing the deleted allele was transferred from *E. coli* S17-1-λpir into parental *Pdd* strain DK32. After conjugation for 48 h on TSA plates prepared with seawater, cells were scrapped off the plate and suspended in TSB-1. Next, 100 μL aliquots of serial decimal dilutions were spread on TCBS agar supplemented with kanamycin to select for *Pdd* clones that have undergone a first recombination event, as growth of *E. coli* donors is inhibited on TCBS agar. Insertion of the suicide vector into the *Pdd* genome by homologous recombination results in kanamycin resistance, and in sucrose sensitivity due to *sacB* gene. Kanamycin resistant colonies were subsequently cultured in TSB-1 without antibiotic selection, and serial decimal dilutions were spread on TSA-1 plates supplemented with sucrose (15% (wt/vol)) to select for a second recombination event. Reisolated colonies were tested by PCR using primer pair scrA-mutant-test (Table 3) to identify recombinants in which the mutant allele of the *scrA* gene replaced the parental allele. This led to mutant strain DK32 *ΔscrA* (SSS165) (Table 2).

### 2.5. Growth Assays with Sucrose as a Carbon Source

Parental DK32, and DK32 *ΔscrA* strain were streaked on a TSA-1 plate and incubated overnight at 25 °C. A loopful of cells was resuspended in phosphate buffered saline (PBS) to achieve an optical density of 0.3 at 600 nm (OD_600_:0.3). For the assay, 1 μL of the bacterial cell suspensions was aliquoted into the wells of a 96-well plate containing 100 μL of M9 minimal medium [47] supplemented, when necessary, with 0.2% (wt/vol) Casamino Acids (Thermo Fisher Scientific Inc, Waltham, MA, USA) (CM9), and with a sugar (0.5% glucose or 2% sucrose (wt/vol)) depending on the aim of the experiment. Final concentration of NaCl was adjusted to 1% in all the assays. For each assay, OD_600_ values were recorded every 10 min for 20 h. This experiment was automated using the spectrophotometer Epoch2 microplate reader (BioTek, Winooski, VT, USA). The 96-well plates were continuously incubated at 25 °C in the plate reader with shaking. Three replicates were performed per assayed condition and strain. Mean values are reported and error bars represent the standard deviations.

### 2.6. Construction of a Transcriptional Fusion of scrA Promoter to a lacZ Reporter Gene, and β-Galactosidase Assays

The putative *scrA* gene promoter was PCR amplified with primer pair scrA-promoter (Table 3) and fused to a promotorless *lacZ* gene in the low-copy-number reporter plasmid pHRP309. The plasmid with the transcriptional fusion construct p*scrA*::*lacZ* (pSSS250), was mobilized from *E. coli* S17-1-λpir into *Pdd* parental strain DK32 by conjugation. After conjugation for 24 h on TSA plates prepared with seawater, cells were scrapped off the plate, suspended in TSB-1, and 100 μL aliquots of serial decimal dilutions were spread on TCBS agar supplemented with gentamicin (resistance provided by pHRP309). DK32 transformants harboring pSSS250 plasmid (DK32 p*scrA*::*lacZ*) were grown in CM9 medium supplemented either with 1% glucose or with 1% sucrose, and β-galactosidase activities were measured as previously described and expressed in Miller units [48]. Three independent experiments with 3 replicates each were conducted. Mean values are reported and error bars represent the standard deviations. The statistical analysis of the gene expression data was carried out with Mann–Whitney test.

### 2.7. Comparative Genomics and Molecular Phylogeny Analyses

Comparative genomics analysis to search for genes specific of Scr+ strains was conducted with RAST [49]. Pfam database was used for predictions of protein domains [50]. Easyfig v.2.2.3. [51], was used for comparative analysis and visualization of gene architecture of *scr* clusters of *Photobacterium* and *Vibrio* species. The Genbank accession numbers of *Vibrio* and *Photobacterium* genomes used in the comparative genomics and in the phylogenetic analyses are listed in Table 4. A phylogenetic tree of 23 *Pdd* complete genomes was constructed using the guide tree obtained by MAUVE genome alignment program (progressive Mauve option) [52,53].

The species tree was generated using the concatenated amino acid sequences of the proteins encoded by 8 housekeeping genes, *ftsZ* (cell division protein FtsZ), *gapA* (glyceraldehyde 3-phosphate dehydrogenase), *gyrB* (DNA gyrase subunit B), *mreB* (rod shape-determining protein MreB), *recA* (RecA recombinase A), *pyrH* (uridylate kinase, uridine monophosphate kinase), *topA* (DNA topoisomerase I)*,* and *toxR* (transmembrane transcription regulator). These housekeeping sequences were selected on the basis of their demonstrated value for fine-tuned discrimination of taxa within the family *Vibrionaceae* [9,54,55]. The sucrose genes tree was constructed using the concatenated amino acid sequences of the proteins encoded by the 4 sucrose cluster genes *scrRAKB*. The Mesquite 3.61 program was used to concatenate the protein sequences [56].

Evolutionary analyses were conducted in MEGA X [57]. The evolutionary history of the strains was inferred using the Neighbor-Joining method [58]. The percentage of replicate trees in which the associated taxa clustered together in the bootstrap test (1000 replicates) is shown next to the branches. The evolutionary distances were computed using the Maximum Composite Likelihood method [59], and are in the units of the number of base substitutions per site.

### 2.8. Database Submission

The draft genome sequences of the 7 *Pdd* strains determined in this study are available from Genbank under accession numbers listed in Table 5.

## 3. Results

### 3.1. Identification of Two Different Genetic Variants of a Four Gene Cluster Encoding Functions for Sucrose Uptake and Catabolism in Pdd Strains

**Figure 1:**
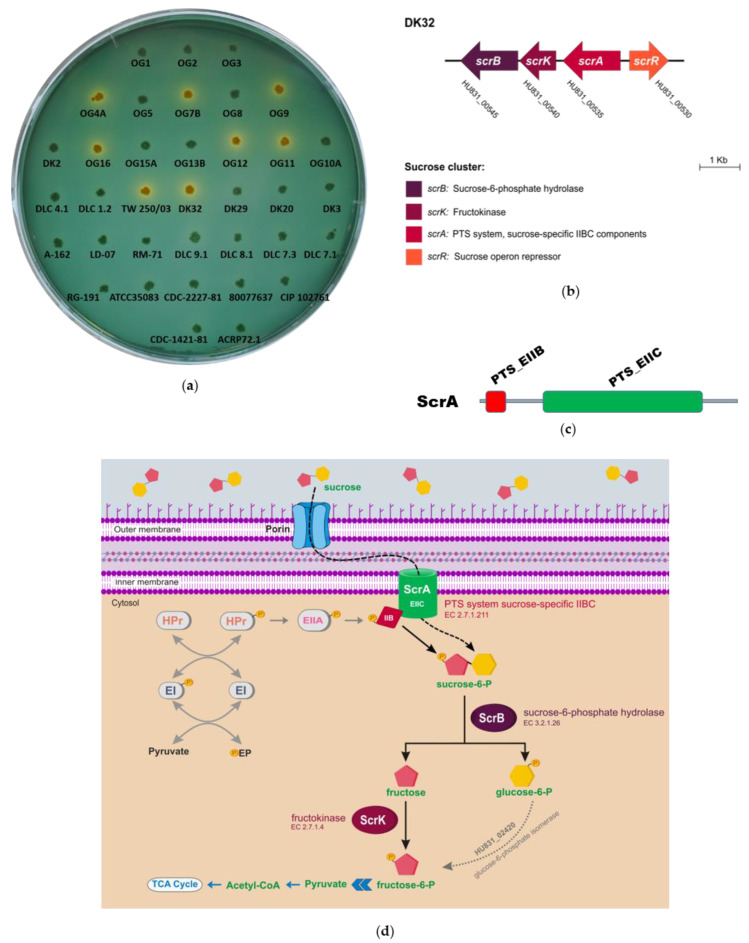
(**a**) Sucrose fermentation phenotypes of 36 *Pdd* strains cultured on thiosulfate-citrate-bile salts-sucrose agar plates (TCBS) agar. Sucrose-fermenting strains (Scr^+^) form yellow colonies whereas non-sucrose fermenters (Scr^−^) form green colonies; (**b**) *scr* cluster of *Pdd* strain DK32 identified in this study, depicting the conserved gene architecture in this subspecies; (**c**) Pfam domain prediction for the *Pdd* ScrA protein, showing the presence of the two domains PTS_EIIB and PTS_EIIC within the same protein; (**d**) A model for sucrose utilization pathway in *Pdd*.

**Figure 2:**
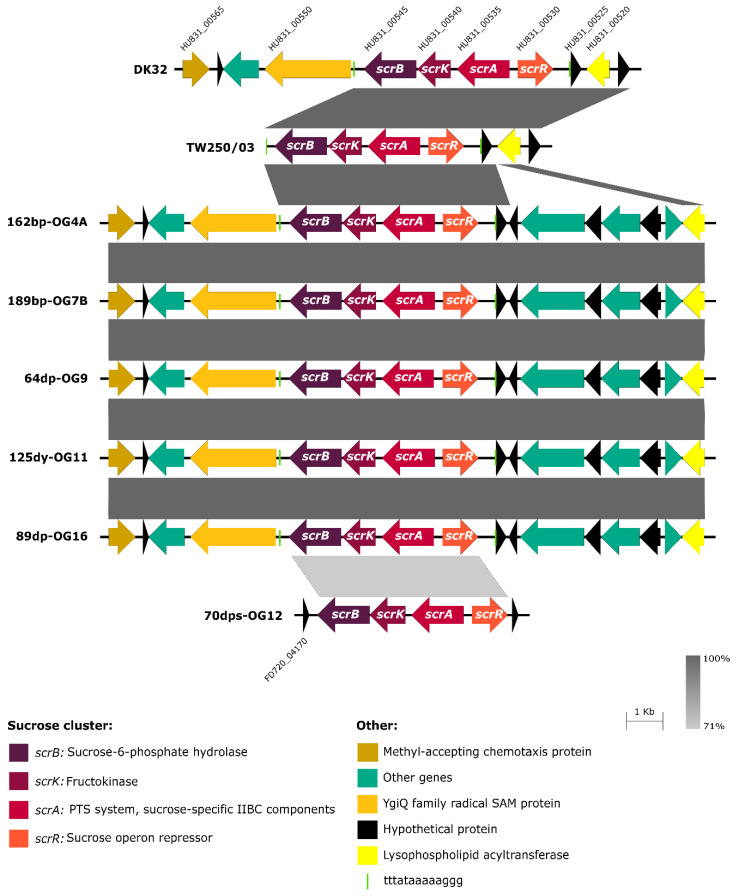
Representation of the *scr* clusters identified in the eight sucrose-degrading *Pdd* strains characterized in the present study, depicting the majority version 1, and the version 2 unique to strain 70dps-OG12. The nucleotide sequence of the cluster of strain 70dps-OG12 differs in 30% from the clusters of the other 7 strains. The genetic context revealed to be highly conserved in 5 out of the 8 strains, with some differences in the context of DK32. For 70dps-OG12 and TW250/03 the flanking regions could not be identified with precision due to difficulties in draft genome assembly, likely motivated by the existence of repeated sequenced regions. With the exception of 70dps-OG12, we identified a duplicated 13-mer sequence (TTTATAAAAAGGG) flanking the *scr* cluster in all the strains, that is denoted by vertical green bars.

In order to study sucrose utilization in this marine pathogen, we screened a collection of 36 *Pdd* isolates from diverse geographical origins and isolation sources, for their ability to utilize sucrose on the selective and differential medium TCBS. This screening revealed that 8 strains grew as yellow colonies, thus exhibiting a Scr^+^ phenotype (Figure 1a; Table 1). To gain an insight into the genetic basis of sucrose utilization, we here obtained the de novo genome sequences of 5 Scr^+^ strains, namely DK32, TW250/03, 162bp-OG4A, 189bp-OG7B and 125dy-OG11, and 2 Scr^−^ *Pdd* isolates, CDC-1421-81 and 82dy-OG8. The general features of the 7 *Pdd* genomes sequenced in the present study are described in Table 5. In addition, we retrieved from GenBank database the genomes of 3 Scr^+^ strains (64bp-OG9, 70dps-OG12, and 89dp-OG16) that have been sequenced in our laboratory in a recent study focused on the presence of large multidrug resistance plasmids in this subspecies [26] (Table 1), as well as 13 genomes of Scr^−^ *Pdd* strains obtained in previous studies in our laboratory (Table 1). *Pdd* genomes were compared using RAST tool [49], searching for genes present in Scr^+^ strains and absent from Scr^−^ strains. Interestingly, the comparative genomics analysis unveiled two distinct versions of a 4-gene *scr* cluster, and the versions differed in their nucleotide sequence in a 30% (Figure 2). The majority version, hereafter version 1, was present in all the Scr^+^ strains except in OG12, that contained the version 2 of the cluster instead. The locus tags of the four genes comprising the *scr* cluster in each of the 8 Scr^+^ genomes are detailed in Table 6. These genes were organized in two divergently transcribed putative operons (Figure 1b and Figure 2). On the one side, *scrR* encodes a putative sucrose operon repressor of the LacI family. The other three genes are transcribed from the opposite strand, and encode, based on their similarity to the *V. alginolyticus* and *V. cholerae* sucrose utilization genes, a PTS system sucrose-specific IIBC component (EC 2.7.1.211) (*scrA*), a fructokinase (EC 2.7.1.4) (*scrK*), and a sucrose-6-phosphate hydrolase (EC 3.2.1.26) (*scrB*), respectively [14,15,16,18].

Protein domain predictions by Pfam database, and similarity search analysis by BLAST, indicated that the *Pdd scrA* gene encodes a EIIBC-domain containing protein, of the phosphoenolpyruvate-dependent phosphotransferase system (PTS) (Figure 1c). According to the in silico analysis of these four genes and their predicted protein products, it is hypothesized that *Pdd* utilizes sucrose via the PTS mechanism, following the scheme depicted in Figure 1d. The conserved PTS system is one of the most common mechanisms for high affinity uptake of sugars in bacteria. It consists of a phosphotransfer cascade that catalyzes the transfer of a phosphate group from phosphoenolpyruvate (PEP) to a carbohydrate substrate concomitant with its transport into the cytoplasm [60,61]. The PTS system includes three major components: Enzyme I (EI), EII (EIA-C) and the histidine-containing phosphocarrier protein (HPr). EI and HPr are shared by all PTS transporters. EI autophosphorylates in the presence of PEP and transfers the phosphate group to HPr. Subsequently, HPr passes the phosphoryl group to a sugar-specific EIIA protein, EIIA passes it to its cognate EIIB, and the latter transfers the phosphoryl group to a sugar bound to the cognate, inner membrane-spanning EIIC element, facilitating sugar import. Thus, the EII complexes consist of three domains, EIIA, EIIB, and EIIC, that can either be part of the same protein (an EIIABC protein), or can be encoded by distinct proteins. The EIIC domain defines the carbohydrate specificity within these transport systems [60]. Once sucrose is taken up by the EIIC component of ScrA, it yields intracellular sucrose-6-phosphate that is hydrolyzed to glucose-6-phosphate and fructose by the sucrose-6-phosphate hydrolase (*scrB*). Fructose is phosphorylated to fructose-6-phosphate by the fructokinase encoded by *srcK*, and the two monosaccharides would enter the glycolytic pathway (Figure 1d).

On the light of the comparative genomics of 23 *Pdd* strains for which genome sequences are available (Table 1), there is a 100% correlation between presence of the *scr* cluster, either version 1 or version 2, and the ability to ferment sucrose on TCBS. A previous study reported that a naturally-occurring, single nucleotide insertion in *scrA* gene rendered *V. cholerae* strain IEC224 unable to ferment sucrose [19]. We thus wanted to assess whether the Scr^−^ phenotype of *Pdd* strains could be due in some instances to point mutations in the sucrose operon. To this aim we selected 13 *Pdd* strains which are phenotypically Scr^−^ and whose genome sequences are not available (Table 1), and conducted a PCR screening with two primer pairs targeted to sequences conserved in the *scrB* and *scrR* genes of the two versions of *scr* clusters described in this study. As a result, it was found that all the Scr^−^ isolates tested negative in the PCR tests, whereas all the Scr^+^ strains tested positive for *scrB* and *scrR* genes (Table 1).

The genetic context upstream and downstream the *scr* cluster was found to be highly conserved in six of the eight Scr^+^ strains (Figure 2), where this cluster is invariably flanked by genes encoding a YgiQ family protein (locus HU831_00550) and a hypothetical protein (locus HU831_00525) (loci tags refer to the DK32 draft genome). The gene homologous to HU831_00550 in the closed genome sequence of the *Pdd* type strain CIP102761 (locus VDA_001759) maps to chromosome I, which suggests that the version 1 of *scr* cluster is chromosome I-borne in *Pdd*. For strains 70dps-OG12 and TW250/3 the flanking regions could not be identified with precision due to gaps in draft genome assembly, likely caused by the existence of repeated sequences, as insertion sequence elements. In any case, the context of sucrose genes in 70dps-OG12 was unrelated to the context of the other 7 Scr^+^ strains. The observation of a highly conserved context in most Scr^+^ strains suggest that the *scr* cluster was inserted into the genome by means of some mechanism of DNA recombination. Notably, a duplicated 13-mer sequence (TTTATAAAAAGGG) was found flanking both sides of the *scr* cluster in all the strains with the exception of 70dps-OG12 (Figure 2).

### 3.2. Deletion of scrA in the scr^+^ Strain DK32 Causes Green Colonies on TCBS Medium, and Abolishes Growth with Sucrose as Carbon Source

**Figure 3:**
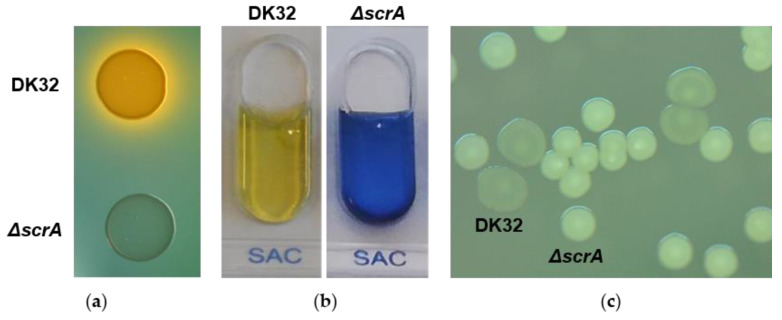
Phenotypical analysis of the *scrA* mutant in *Pdd* DK32. Deletion of *scrA* produces green colonies on TCBS agar (**a**), yields a negative result in the sucrose fermentation test in API-20E (**b**), and causes changes in colony morphology compared to the parental strain when grown on TSA agar plates supplemented with 15% sucrose (**c**).

As described above, *scrA* encodes a EIIBC-domain containing protein with a predicted role in sucrose utilization. In order to investigate this, a non-polar, unmarked deletion mutant for *scrA* gene was constructed in the *Pdd* wild type strain DK32 by allelic exchange, a process that removed 90% of the *scrA* coding sequence without disrupting the reading frame. As a result, it was observed that DK32 *ΔscrA* produced green colonies on TCBS (Figure 3a), suggesting that this mutant is unable to transport sucrose into the cell. In addition, DK32 *ΔscrA* produced a negative result for sucrose fermentation in the API-20E gallery, which is routinely used in laboratories for the identification of fish pathogenic bacteria (Figure 3b). Unexpectedly, mutation of *scrA* caused a change in the colony phenotype compared to the wild type colonies, when the strains were cultured on TSA-1 supplemented with 15% (wt/vol) sucrose, the sugar concentration used to select for a second recombination step during the allelic exchange process (see methods). DK32 wt colonies appeared flat and translucent while DK32 *ΔscrA* colonies appeared convex and whitish (Figure 3c). We thus hypothesized that the presence of the sucrose fermenting gene cluster in the bacterial genome might be responsible for causing such phenotype when the strain is growing in presence of high concentrations of sucrose. In order to test this, we analyzed the colony morphologies of the naturally Scr^+^ and Scr^−^ strains 89dp-OG16 and LD-07, respectively. Strains containing the *scr* cluster yielded flat and translucent colonies, whereas Scr^−^ strains yielded convex and whitish colonies. The colony phenotypes of Scr^+^ and Scr^−^ strains were indistinguishable on TSA-1 without added sucrose (Supplementary Figure S1).

**Figure 4:**
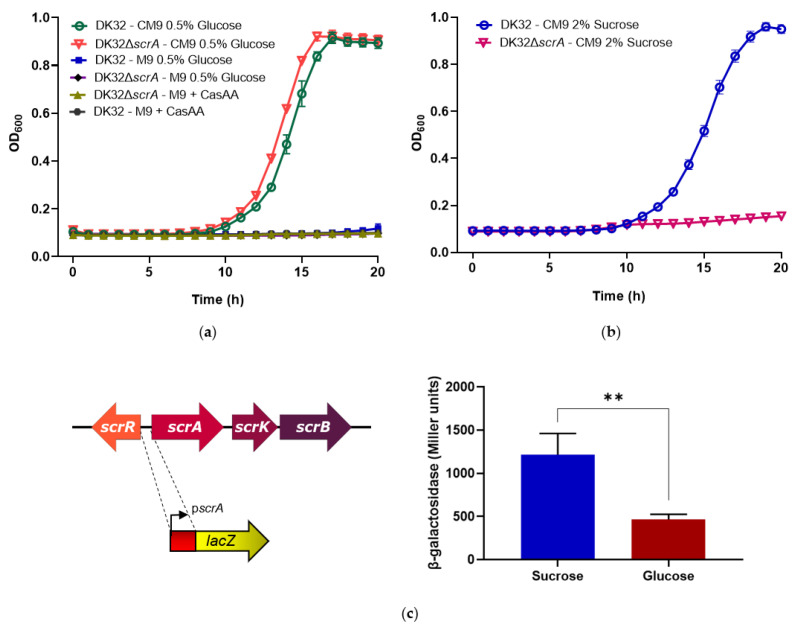
(**a**) Growth curves of *Pdd* DK32 parental and *ΔscrA* strains in minimal medium M9 and in M9 supplemented with casamino acids (CasAA) (CM9), with or without glucose. *Pdd* is unable to grow in minimal medium unless casamino acids and sugar (glucose) are provided. No differences in growth are detected between parental and *ΔscrA* strains; (**b**) Substitution of 0.5% glucose by 2% sucrose in CM9 sustains growth of DK32 parental strain, whereas *ΔscrA* mutant growth is abolished; (**c**) A transcriptional fusion of *scrA* promoter to a reporter *lacZ* gene reveals that promoter activity is 2.5-fold upregulated when sucrose substitutes glucose as carbon source. Mean values ± SE; *n* = 3; **, *p* ≤ 0.01. Statistical significance was determined by an unpaired two-tailed Mann–Whitney test.

We next studied how deletion of *scrA* impacted *Pdd* DK32 growth with sucrose as a carbon source. To this aim, it was first necessary to optimize the conditions for culturing *Pdd* in minimal medium. It is pertinent to highlight that no detectable growth was achieved neither by the parental strain nor by the *ΔscrA* mutant when cultured in minimal medium M9 with glucose 0.5%, suggesting that *Pdd* DK32 cannot efficiently synthesize all the necessary amino acids in a mineral minimal medium with glucose as carbon source (Figure 4a). Similarly, the minimal medium M9 supplemented with casamino acids as sole carbon source in the absence of glucose, did not support growth within 20 h (Figure 4a), suggesting that *Pdd* does not efficiently use amino acids as carbon source, and needs a sugar for optimal growth. In support of these hypotheses, growth of DK32 and DK32 *ΔscrA* strains was completed to the stationary phase within 20 h when casamino acids were added to M9 with glucose 0.5% (CM9 medium) (Figure 4a). These results demonstrate that deletion of *scrA* does not cause a fitness cost in the growth of *Pdd* with glucose as carbon source in presence of casamino acids. However, when 2% sucrose was added to CM9 medium in substitution of glucose, the DK32 *ΔscrA* mutant was drastically impaired for growth, whereas the parental strain achieved full growth values as with glucose (Figure 4b).

We also analyzed whether the transcriptional activity of the promoter upstream *scrA* was affected by growth in presence of sucrose. To this aim, the *scrA* promoter sequence (p*scrA*) was fused to a promoterless *lacZ* gene in plasmid pHRP309, that was further mobilized to *Pdd* parental strain DK32, yielding DK32 p*scrA*::*lacZ*. This indicator strain was cultured in CM9 medium supplemented with either 1% sucrose or 1% glucose. The measure of β-galactosidase activities demonstrated that *scrA* promoter exhibited basal transcription levels in presence of glucose as carbon, and promoter activity was increased 2.5-fold in the presence of sucrose as carbon source (Figure 4c). Collectively, the growth assay data and transcriptional fusion analysis, demonstrate that *scrA* is necessary for sucrose utilization as carbon source in *Pdd* and its transcription is increased in presence of sucrose.

### 3.3. Scr Clusters Are Inserted into Putative Hot-Spots for DNA Acquisition in Pdd Genomes

**Figure 5:**
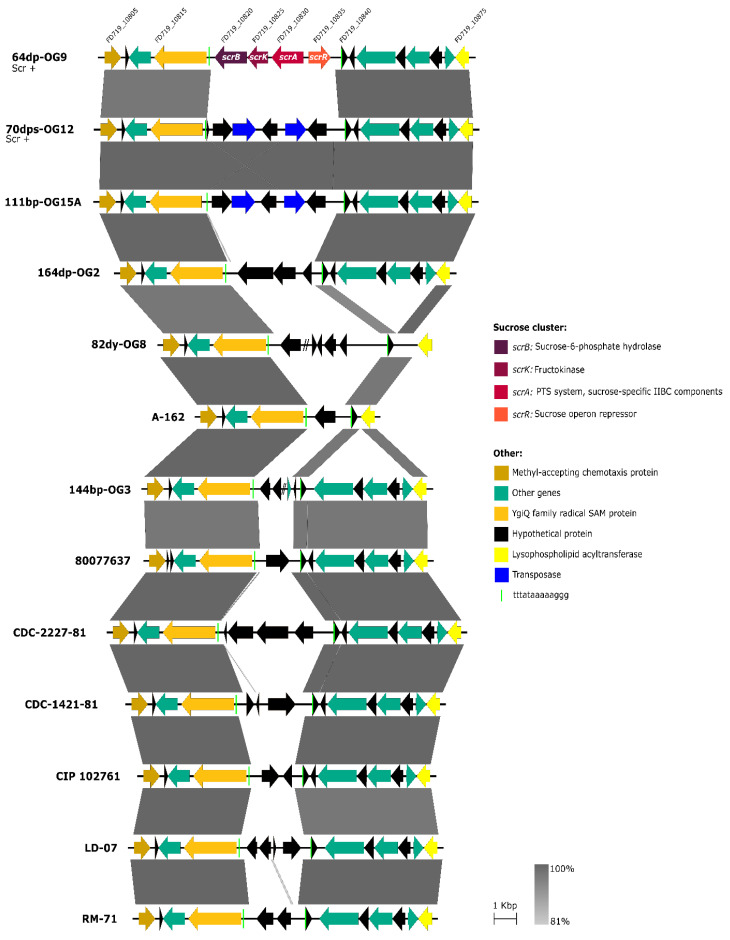
Version 1 *Pdd scr* clusters are inserted within a genome region that shows high genetic plasticity among *Pdd* strains. The *scr* cluster of strain 64bp-OG9 is shown as reference. Note that the 11 depicted Scr^−^ strains, as well as Scr^+^ strain 70dps-OG12 (that harbors a *scr* cluster in another genomic location) each contain a unique gene repertoire in this genomic region, that is proposed to be a hot-spot for recombination of horizontally acquired DNA. The variable DNA is in most cases flanked by a direct repeat of the 13-mer sequence TTTATAAAAAGGG.

We hypothesized that the genome region flanked by the genes encoding the YgiQ family protein and the lysophospholipid acyltransferase, i.e., the DNA region harboring the *scr* cluster in the majority of Scr^+^ *Pdd* strains, represents a hot-spot for events of DNA acquisition and loss. In order to gain an insight into this, we conducted a comparative analysis of the gene composition within this putative hot-spot in eleven Scr^−^ *Pdd* strains, which included two strains newly sequenced in the present study (CDC-1421-81 and 82dy-OG8) and 9 additional Scr^−^ strains whose draft genomes were retrieved from GenBank database (Table 1). We found that 70dps-OG12, the Scr^+^ strain whose context of the *scr* cluster remains unknown, contained a number of hypothetical proteins and transposases in this region, and was identical to the gene composition of the homologous region in the Scr^−^ strain 111bp-OG15A (Figure 5). Notably, analysis of the 11 Scr^−^ genomes unveiled the existence of 11 unique gene combinations, i.e., each strain contained genes with no counterparts in the other *Pdd* genomes. These observations provide strong evidence that this genome region is highly prone to events of DNA acquisition and can be considered as a recombination hot-spot for acquisition of DNA. Of note, the duplicated 13-mer sequence TTTATAAAAAGGG was found in most strains at exactly the same point (Figure 5).

### 3.4. Scr Clusters Occur in Different Genetic Lineages of Pdd

**Figure 6:**
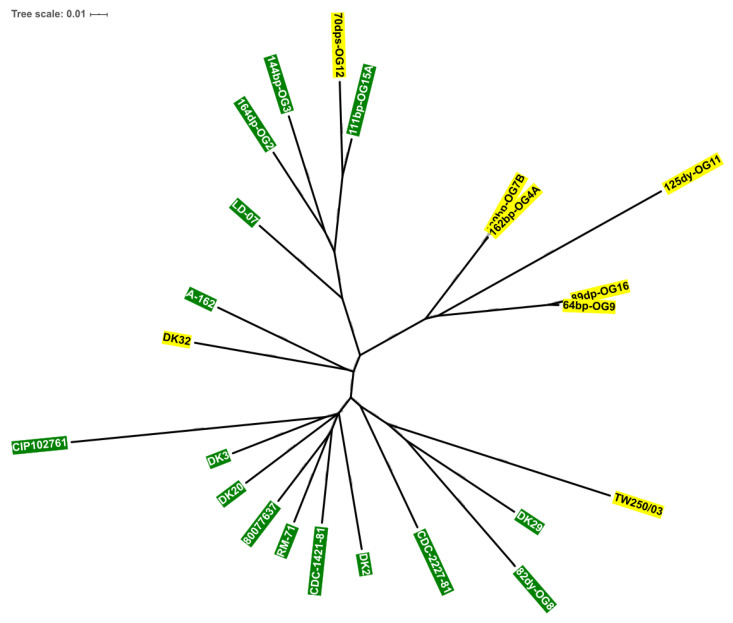
Dendrogram of Scr^+^ (yellow) and Scr^−^ (green) *Pdd* strains based on the MAUVE genome alignment program, showing that *scr* clusters occur in different genetic lineages of this subspecies.

In this study, 8 *Pdd* strains contained a *scr* gene cluster. Of these, six were isolated from European seabass in the Black Sea [30], one from diseased rainbow trout in Denmark [24,37] and one from gilthead seabream from unknown geographical origin (Table 1). In order to ascertain whether the sucrose positive strains conform a clonal entity or whether the *scr* clusters have been acquired by different genetic lineages of *Pdd*, we carried out a comparative analysis of 23 complete *Pdd* genomes. As shown in Figure 6, five Scr^+^ strains isolated from the Black Sea group together within a clade, and among these, the pairs OG7B/OG4A and OG9/OG16 likely correspond to two clonal lines respectively. However, 125dy-OG11 is distantly related from the aforementioned four strains, and clearly represents an evolutionary line that has diverged from the other strains. Of note, the three remaining Scr^+^ strains are unevenly distributed with other Scr^−^ genomes in different branches of the phylogenetic tree. It is noticeable that strain DK32, isolated in the coast of Denmark in 2006, harbors a *scr* cluster 99.9% identical at the nucleotide sequence level to the *scr* cluster of 125dy-OG11, isolated in the Black Sea in 2011, despite these two strains being distantly located in the phylogenetic tree (Figure 6). All these results provide strong evidence that the Scr^+^ isolates are not clonal derivatives (with the exception of the pairs OG7B/OG4A and OG9/OG16), and support the hypothesis that *scr* clusters have been acquired by horizontal transfer by different genetic lineages of *Pdd*, as a result of independent events of DNA acquisition.

### 3.5. Incongruences between the Species Tree and the Sucrose Genes Tree Reveal Extensive Horizontal Transfer of scr Genes among Species of Vibrio and Photobacterium

**Figure 7:**
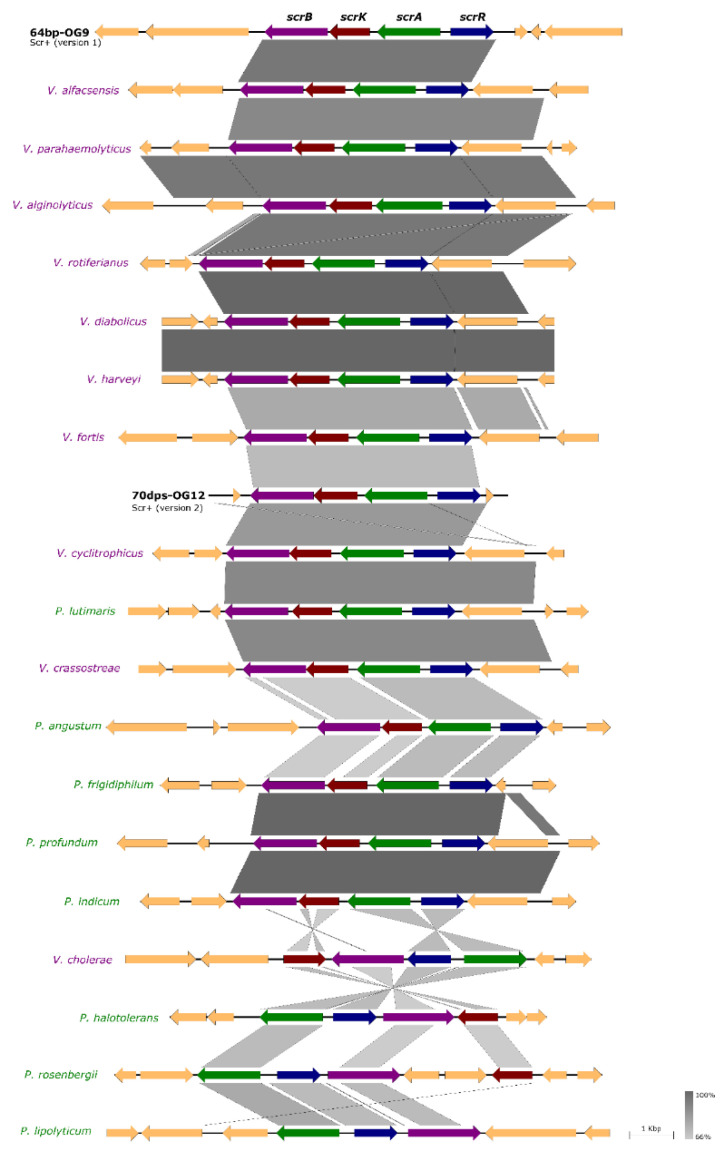
Comparative analysis of the sucrose degradation gene clusters in different species of *Vibrio* and *Photobacterium*, including the *Pdd* strains 64bp-OG9 (representative of version 1 of *scr* cluster) and 70dps-OG12 (version 2).

The high percentages of sequence identity and of operon architecture in *scr* cluster genes among 7 of the 8 *Pdd* strains analyzed in the present study, clearly indicate a common evolutionary origin of sucrose cluster genes in these strains. In addition, the observation that the *scr* cluster in strain 70dps-OG12 exhibits 30% sequence divergence with respect to the other 7 *Pdd* strains, suggests that *scr* clusters have been gained in this subspecies by horizontal gene transfer from different donor species. Although sucrose utilization is a widespread trait in many species within the family *Vibrionaceae*, the phylogenetic relationships among sucrose utilization clusters from different species remains largely uncharacterized. Here, we conducted a Genbank database search to retrieve gene clusters encoding sucrose utilization genes in species of *Photobacterium* and *Vibrio* (Accession numbers are listed in Table 4) that were homologous to the *Pdd* clusters. A schematic analysis of the two representative *Pdd* clusters (64bp-OG9 and 70dps-OG12) and of 18 *Photobacterium* and *Vibrio* clusters revealed that gene architecture is largely conserved, with the exception of *V. cholerae*, *P. halotolerans*, *P. rosenbergii,* and *P. lipolyticum* that showed different gene arrangements (Figure 7).

**Figure 8:**
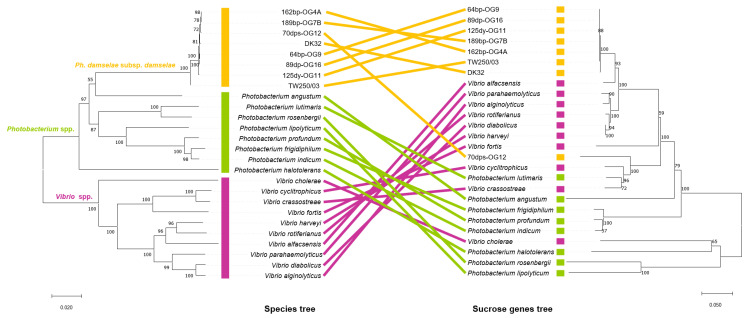
Comparison of the sucrose operon tree (right side) with the species tree (left side) for Scr^+^ *Pdd* strains, and additional *Photobacterium* and *Vibrio* species. The sucrose operon tree was constructed based on the amino acid sequence alignment of the concatenated proteins ScrB, ScrK, ScrA, and ScrR. The species tree was constructed based on the amino acid sequence alignment of 8 concatenated proteins encoded by housekeeping genes *ftsZ*, *gapA*, *gyrB*, *mreB*, *recA*, *pyrH*, *top,* and *toxR*. Species and strains are connected with different colors in both trees: *Pdd* strains (orange), *Photobacterium* spp. (green) and *Vibrio* spp. (purple). The noticeable incongruence between sucrose operon and species tree indicates that sucrose genes of *Pdd* are evolutionarily closely related to sucrose clusters of *Vibrio* species, while more distantly related to the sucrose clusters found in the majority of *Photobacterium* species. Bootstrap support values are displayed on the nodes.

In order to determine the evolutionary history of sucrose uptake and catabolism genes, we reconstructed the phylogeny of ScrB, ScrK, ScrA and ScrR proteins among *Photobacterium* and *Vibrio* species. Proteins representing homologues within these two genera were aligned using ClustalW and phylogenetic trees were constructed by the neighbor-joining method using MEGA X [57]. This analysis demonstrated that the *scr* genes of 7 out of the 8 *Pdd* strains (version 1 cluster), are closely related to the clusters found in *Vibrio* species. On the contrary, the cluster of *Pdd* 70dps-OG12 (version 2 cluster) is related to clusters found in species of the genus *Photobacterium* and shares the same branch with *V. cyclitrophicus* and *V. crassostreae* (Figure 8). These observations clearly suggest that *scr* clusters have undergone horizontal gene transfer among species of the two genera. In order to clarify this, we conducted in parallel a phylogenetic analysis of the same *Photobacterium* and *Vibrio* species, by analyzing the concatenated amino acid sequences of eight housekeeping genes conserved in all the species, and producing a “species tree” that was compared to the “sucrose genes tree”. The analysis of these housekeeping genes generated a phylogenetic tree with three distinct clades that include the 8 *Pdd* strains, the rest of *Photobacterium* species, and the *Vibrio* species, respectively. The noticeable incongruence between sucrose operon tree and species tree clearly indicates that sucrose genes found in 7 *Pdd* strains (version 1 cluster) are closely related to clusters of *Vibrio* species, being *V. alfacsensis* the closest relative, while they are more distantly related to the clusters of *Photobacterium* species. This suggests that *Pdd* sucrose cluster version 1 may have been acquired by *Pdd* strains from a *Vibrio*-like donor. The *scr* cluster of *Pdd* 70dps-OG12 (version 2) has a different evolutionary history than the other 7 *Pdd* clusters, and is more similar to both *Vibrio* and *Photobacterium* clusters. Notably, the close evolutionary distance between sucrose clusters of *P. lutimaris* and *V. crassostreae* contrasts with the placement of these two species in two different clades in the species tree. All these observations provide strong evidence that horizontal gene transfer of sucrose genes is extensive among *Photobacterium* and *Vibrio* species.

## 4. Discussion

*Pdd* (formerly known as *V. damsela*), is an important and emerging pathogen for marine animals and for humans [22]. An early review on pathogenic *Vibrio* species already highlighted that a 5% of *Pdd* isolates were sucrose fermenters on TCBS agar [27]. Since then, various studies have unveiled the existence of Scr^+^ *Pdd* isolates at varying frequencies. An extensive study that analyzed 71 *Pdd* isolates from fish in Spain, reported that 4% were sucrose-fermenters [28]. In another study with *Pdd* strains from disease outbreaks in marine rainbow trout, one isolate out of 31 tested positive for sucrose fermentation [24]. A study reported the simultaneous isolation on a TCBS agar plate of yellow and green *Pdd* colonies from different organs of the same fish [29]. Notably, in a recent study, of 14 *Pdd* strains isolated from diseased seabass in the Turkish coast of the Black Sea, 6 were able to ferment sucrose [30], which accounts for 40% of strains positive for this trait. Thus, albeit sucrose fermentation can be considered as an infrequent phenotype in this subspecies, the intraspecies variability of this metabolic trait needs to be taken into consideration, in order to avoid misidentification of potential *Pdd* strains in veterinary, clinical and environmental studies.

Even though sucrose fermentation tests are routinely used for identification and classification of species of the *Vibrionaceae*, the genetic basis of sucrose utilization in species of this family has received scarce attention. Homologues of the *scr* cluster genes described in the present study in *Pdd*, were reported for the first time in *V. alginolyticus* in pioneering studies in the family *Vibrionaceae* [14,15,16]. Later, different investigations reported some data on sucrose utilization genes in *V. cholerae* [17,18,19,20] whereas, to the best of our knowledge, no functional and evolutionary analyses have been conducted in species of the genus *Photobacterium* so far. In this study, we describe two variants of a gene cluster that is required for the utilization of sucrose by *Pdd*. Mutational analysis has demonstrated that *scrA* gene encoding the predicted EIIBC component of the PTS sucrose transport system is essential for sucrose utilization as carbon source by *Pdd*. In a previous study, the systematic genetic dissection of PTS systems in *V. cholerae* demonstrated that single deletion of VCA0563, a gene homologous to the *Pdd scrA*, was sufficient to abolish sucrose utilization [20], indicating that no redundant PTS functions existed in the *V. cholerae* genome capable of conferring the ability to take up sucrose. In contrast, *V. cholerae* showed to encode redundant PTS functions for the uptake of glucose, mannose and fructose [20]. We have here found that single deletion of *scrA* abolished sucrose utilization in *Pdd* DK32, demonstrating that this strain does not encode redundant PTS functions for the uptake of this disaccharide.

The *scr* clusters in some species of *Enterobacteriaceae* are known to be negatively controlled by the repressor encoded by *scrR* gene and thus genes encoding enzymes for sucrose catabolism are inducible by presence of sucrose in the medium [62,63]. The *Pdd scr* cluster also contains a homologue of *scrR* gene, but so far, no experimental evidence on the sucrose-inducible nature of *scr* clusters in the *Photobacterium* genus was available. We here have shown that growth of *Pdd* DK32 with sucrose as carbon source increased in 2.5-fold the transcriptional activity of the *scrA* promoter compared to growth in presence of glucose as carbon source, suggesting that sucrose metabolism in *Pdd* is subjected to a negative regulation similar to that described in some enterobacteria.

It was observed that presence of *scr* cluster genes imparted a unique colony phenotype to *Pdd* strains when grown in presence of high sucrose concentrations (15%). The mechanisms underlying this phenotype await further and promising studies in *Pdd*. Interestingly, a recent study pointed out that the *V. cholerae* VCA0653 gene encoding the sucrose-specific PTS component ScrA, was 30-fold downregulated on the phase variation switch from wild-type-colony phenotype to a rugose-colony phenotype, associated with advanced biofilm architecture [64]. This suggests that the ability to utilize sucrose would impair biofilm formation, and thus transition to a rugose phenotype correlates with a strong downregulation of sucrose uptake. Similarly, *Lactobacillus* strains grown on sucrose were found to produce dextrane, and it influenced self-aggregation and biofilm formation [65].

Carbohydrates constitute a main carbon source in bacteria [66]. Horizontal gene transfer enables bacteria to acquire new gene clusters that provide the recipient with novel metabolic activities to confront ecological changes [67,68]. Acquisition by horizontal transfer of genes for transport and catabolism of carbohydrates in members of the *Vibrionaceae* is being increasingly reported, as is the case of genes for cellobiose utilization [69], for degradation of algal polysaccharides [8], and genes of D-galactose metabolism [11], among others. Sucrose utilization clusters are not an exception to this, and previous studies have pointed out that sucrose gene clusters have been extensively exchanged among *Eubacteria* [21]. In the present study, the incongruence of phylogenies based on 8 representative housekeeping genes on the one side, and on the four *scr* genes on the other side, clearly revealed that genes for sucrose metabolism have undergone extensive horizontal transfer among genera and species within the family *Vibrionaceae*. Of the two *scr* cluster versions reported in the present study, the majority version 1 is likely chromosome I-borne considering that the conserved genome context upstream and downstream of sucrose genes corresponds to chromosome I genes in well studied Scr^−^ *Pdd* genomes. In addition, the seven *scr* clusters of version 1 are invariably flanked by a duplicated 13-mer sequence that is a putative candidate to play a role in DNA acquisition. A recent study has reported that some regions of the *Pdd* genome constituted hot-spots for DNA acquisition, and such hypervariable regions were flanked by repeated sequences in tandem [24]. Similarly, sucrose utilization genes in enteropathogenic *E. coli* strains were found to be located within variable chromosomal regions rich in repeated sequences dubbed *iap* sequences [70]. The genetic context of version 2 represented by *Pdd* strain 70dps-OG12 is uncertain, and it might be either chromosome- or plasmid borne. It is known that genes for sucrose metabolism in *Enterobacteria* are either chromosome-borne [71,72] or plasmid-borne [73,74,75]. Further studies are prompted in order to ascertain the existence of plasmid-borne *scr* clusters in members of the family *Vibrionaceae*.

The acquisition of sucrose metabolism genes by species of *Vibrionaceae* raises the question about what is the role of sucrose utilization in the ecology of Vibrios. Sucrose is the most abundant disaccharide on earth because of its origin in higher plant tissues. Early studies reported that green microalgae of the genus *Trebouxia* secreted sucrose to the culture medium [76], and nowadays it is widely known that green algae, cyanobacteria and purple bacteria synthesize sucrose [77]. Microalgae and cyanobacteria species accumulate sucrose as compatible osmolyte under osmotic stress, a process that has been mainly studied in freshwater environments [78,79,80,81,82,83]. A recent study reported the production of sucrose by marine species of *Pyrocystis* and *Nannochloropsis* genera [84]. Thus, either secreted by live cells or released upon cell lysis, sucrose is expected to be available as a carbon source in marine ecosystems. In support of this idea, a recent metabolomics study has reported the detection of abundant sugars, which included sucrose and trehalose, in marine ecosystems [85], and sucrose has also been detected as a major nutrient available in seagrass (*Posidonia oceanica*) meadows ecosystems [86]. It is also expected that the digestive tracts of marine herbivores as sea urchins and algae grazers among others, constitute a sucrose-rich niche for bacteria. Of note, a previous study has demonstrated that sucrose is a potent chemoattractant for *Vibrio furnissii* and this chemotaxis was suggested to be dependent on presence of an intact PTS system [87]. Hence, it is hypothesized that the *scr* gene clusters would confer advantage to the bacterial cells to use sucrose available in marine environment niches as a carbon source. Studies to test this hypothesis are currently under way.

## Figures and Tables

**Table 1:** General information, and results of the phenotypic tests for sucrose utilization and of PCR detection of *scr* genes, for the 36 *Pdd* strains used in this study.

Strain	Isolation Source	Strain Reference	Scr Phenotype ^a^	Presence of Sucrose Genes ^b^	Genbank Acc. No. ^c^	Genome Reference
154dp-OG1	European sea bass, Turkey	[30]	−	−	NA	NA
164dp-OG2	European sea bass, Turkey	[30]	−	−	VAUU00000000	[26]
144bp-OG3	European sea bass, Turkey	[30]	−	−	VAND00000000	[26]
162bp-OG4A	European sea bass, Turkey	[30]	+	+	JABWTP000000000	This study
158dp-OG5	European sea bass, Turkey	[30]	−	−	NA	NA
189bp-OG7B	European sea bass, Turkey	[30]	+	+	JABXOP000000000	This study
82dy-OG8	European sea bass, Turkey	[30]	−	−	JABXOQ000000000	This study
64bp-OG9	European sea bass, Turkey	[30]	+	+	VANE00000000	[26]
156dp-OG10A	European sea bass, Turkey	[30]	−	−	NA	NA
125dy-OG11	European sea bass, Turkey	[30]	+	+	JACFTX000000000	This study
70dps-OG12	European sea bass, Turkey	[30]	+	+	VANF00000000	[26]
164dpbuy-OG13B	European sea bass, Turkey	[30]	−	−	NA	NA
111bp-OG15A	European sea bass, Turkey	[30]	−	−	VANG00000000	[26]
89dp-OG16	European sea bass, Turkey	[30]	+	+	VANH00000000	[26]
CIP 102761	Damselfish, United Estates	[32]	−	−	ADBS00000000	Unpublished
TW250/03	Gilthead seabream	Laboratory collection	+	+	JABXOR000000000	This study
RM-71	Turbot, Spain	[33]	−	−	LYBT00000000	[34]
A-162	Eel, Belgium	NA	−	−	LZFN00000000	[34]
LD-07	Gilthead seabream, Spain	[35]	−	−	LYBU00000000	[34]
DK2	Rainbow trout, Denmark	[24,36]	−	−	PVXF00000000	[24]
DK3	Rainbow trout, Denmark	[24,36]	−	−	PVXG00000000	[24]
DK20	Rainbow trout, Denmark	[24,37]	−	−	PVXH00000000	[24]
DK29	Rainbow trout, Denmark	[24,37]	−	−	PVXI00000000	[24]
DK32	Rainbow trout, Denmark	[24,37]	+	+	JABWTO000000000	This study
CDC-1421-81	Fish, Senegal	[38]	−	−	JABXYE000000000	This study
RG-191	Turbot, Spain	[33]	−	−	NA	NA
ATCC35083	Brown shark, United States	[39]	−	−	NA	NA
CDC-2227-81	Human, United States	[38]	−	−	VZUQ00000000	[40]
80077637	Human, Australia	[23]	−	−	WAEO00000000	[40]
ACRP-72.1	Turbot, Portugal	Laboratory collection	−	−	NA	NA
DLC 1.2	Gilthead seabream, Spain	Laboratory collection	−	−	NA	NA
DLC 4.1	Gilthead seabream, Spain	Laboratory collection	−	−	NA	NA
DLC 7.1	Gilthead seabream, Spain	Laboratory collection	−	−	NA	NA
DLC 7.3	Gilthead seabream, Spain	Laboratory collection	−	−	NA	NA
DLC 8.1	Gilthead seabream, Spain	Laboratory collection	−	−	NA	NA
DLC 9.1	Gilthead seabream, Spain	Laboratory collection	−	−	NA	NA

^a^ Scr phenotype evaluated by growth on TCBS agar: +, Sucrose degrading strain producing yellow colonies on TCBS; −, non-sucrose degrading strain producing green colonies. ^b^ Presence of *scr* cluster genes for those strains whose genome sequence is not available was assessed by PCR. ^c^ NA: Not available.

**Table 2:** Strains and plasmids used and constructed in this study.

Strain or Plasmid	Description ^a^	Reference/Source
**Strains**		
*P. damselae* subsp. *damselae*		
DK32	Isolated from rainbow trout; ferments sucrose (Scr^+^)	[24,37]
SSS165	DK32 *ΔscrA*. Does not ferment sucrose (Scr^−^)	This study
SSS250	DK32 with plasmid pSSS250	This study
*E. coli*		
DH5α	Cloning strain	Laboratory stock
S17-1-λ*pir*	RP4-2(Km::Tn7, Tc::Mu-1) *pro-82* λ*pir recA1 endA1 thiE1 hsdR17 creC510*	[41]
**Plasmids**		
pHRP309	*lacZ* reporter plasmid, *mob* Gm^r^	[42]
pSSS250	pHRP309 with a transcriptional fusion of *scrA* promoter to *lacZ*	This study
pWKS30	Low-copy-number cloning vector; Ap^r^	[43]
pNidKan	Suicide vector derived from pCVD442; Km^r^	[44]

^a^ Gm^r^: Gentamicin resistant; Ap^r^: Ampicillin resistant; Km^r^: Kanamycin resistant.

**Table 3:** Primer pair combinations used in this study.

Name of Primer Pair	Oligonucleotide Sequence ^1^	Amplicon Size (bp)
scrA-1-2	F: 5’-GCTCTAGAGCCATTCGCACAACACTTTG-3’ R: 5’-GCGGATCCGTTCGCTAGATCAGTCAATC-3’	2106
scrA-3-4	F: 5’-GCGGATCCTCAAGGTGCTGCCGCTTTAG-3’ R: 5’-GCGAATTCAGGACCTTTATGCTGCCACG-3’	2122
scrA-mutant-test	F: 5’-GGCTCAGGCATAGTAAACCA-3’ R: 5’-CCGCGATAAATGGGTAACGT-3’	1024
scrA-promoter	F: 5’-GCTCTAGAACATCATGCAGACTCGCCAT-3’ R: 5’-GCGGATCCCTCTTTAGCTACTGCCGGAT-3’	281
scrK	F: 5’-TTACGCGACTCACCTCGACA-3’ R: 5’-ATCGGTCGCGCAGAACAAAC-3’	373
scrB	F: 5’-GACCAAGACTACGATTCACA-3’ R: 5’-ACACTCCCACATGTACCCAA-3’	372

^1^ Underlined sequences denote recognition sites for restriction enzymes.

**Table 4:** GenBank accession numbers of the *Vibrio* and *Photobacterium* genomes whose sucrose degrading cluster genes (*scr* genes) were used in the comparative genomics and phylogenetic analyses in this study.

Species	Accession Number
*Vibrio alfacsensis*	CP032093.1
*V. parahaemolyticus*	QPIY01000005
*V. alginolyticus*	AAPS01000004
*Vibrio rotiferianus*	NZ_KV861318
*Vibrio diabolicus*	CP014133
*Vibrio harveyi*	CP014038
*Vibrio fortis*	NZ_JFFR01000009
*Vibrio cyclitrophicus*	VUKB01000001
*Photobacterium lutimaris*	NZ_SNZO01000003
*Vibrio crassostreae*	NZ_AJZB02000137
*Photobacterium angustum*	NZ_PYOK01000006
*Photobacterium frigidiphilum*	NZ_PYMJ01000001
*Photobacterium profundum*	NZ_PYOD01000001
*Photobacterium indicum*	NZ_PYOC01000002
*V. cholerae*	NZ_VTLI01000001
*Photobacterium halotolerans*	NZ_AULG01000013
*Photobacterium rosenbergii*	NZ_PYMB01000001
*Photobacterium lipolyticum*	NZ_PYMC01000002

**Table 5:** General features of the 7 *Pdd* genomes sequenced in this study.

Attribute	162bp-OG4A (Scr^+^)	189bp-OG7B (Scr^+^)	82dy-OG8 (Scr^−^)	125dy-OG11 (Scr^+^)	DK32 (Scr^+^)	TW250/03 (Scr^+^)	CDC-1421-81 (Scr^−^)
Accession no.	JABWTP000000000	JABXOP000000000	JABXOQ000000000	JACFTX000000000	JABWTO000000000	JABXOR000000000	JABXYE000000000
Genome size (bp)	4,306,101 bp	4,302,857 bp	4,627,325 bp	4,450,948 bp	4,248,331 bp	4,695,503 bp	4,432,211 bp
Contigs	133	127	138	147	106	1649	81
% GC	40.70%	40.70%	40.70%	40.70%	40.60%	39.80%	40.40%
Genes (total)	3809	3807	4096	4089	3750	5089	3916
CDSs	3756	3758	4038	3879	3699	5041	3860

**Table 6:** Locus tags of the *scr* gene clusters in the 8 Scr^+^ *Pdd* genomes analyzed in this study.

Strain	162bp-OG4A	189bp-OG7B	64bp-OG9	125dy-OG11	70dps-OG12	89dp-OG16	DK32	TW250/03
Accession no.	JABWTP000000000	JABXOP000000000	VANE00000000	JACFTX000000000	VANF00000000	VANH00000000	JABWTO000000000	JABXOR000000000
Sucrose operon genes (locus_tag)								
*scrA*	HU985_14400	HVV26_09235	FD719_10830	H3N34_00965	FD720_04185	FD722_11585	HU831_00535	HWA77_17195
*scrB*	HU985_14390	HVV26_09225	FD719_10820	H3N34_00955	FD720_04175	FD722_11575	HU831_00545	HWA77_17185
*scrK*	HU985_14395	HVV26_09230	FD719_10825	H3N34_00960	FD720_04180	FD722_11580	HU831_00540	HWA77_17190
*scrR*	HU985_14405	HVV26_09240	FD719_10835	H3N34_00970	FD720_04190	FD722_11590	HU831_00530	HWA77_17200

## Supplementary Materials

The following are available online at https://www.mdpi.com/2073-4425/11/11/1244/s1, Figure S1: Colony morphologies of Scr^+^ and Scr^−^ *Pdd* strains grown in either plain TSA-1 or in TSA-1 supplemented with 15% sucrose. Strains harboring the *scr* gene cluster form flat and translucent colonies in presence of high concentrations of sucrose, while strains lacking a functional *scr* cluster form convex and whitish colonies. The colony phenotypes of Scr^+^ and Scr^−^ strains are indistinguishable on TSA-1 without added sucrose.
