# Supplementary material for: Diverse Horizontally-Acquired Gene Clusters Confer Sucrose Utilization to Different Lineages of the Marine Pathogen Photobacterium damselae subsp. damselae
Source: Genes (Basel). 2020 Oct 22;11(11):1244. doi: 10.3390/genes11111244 (PMC7690375; doi:10.3390/genes11111244)
Supplement: Supplementary file 1 [file genes-11-01244-s001.pdf]

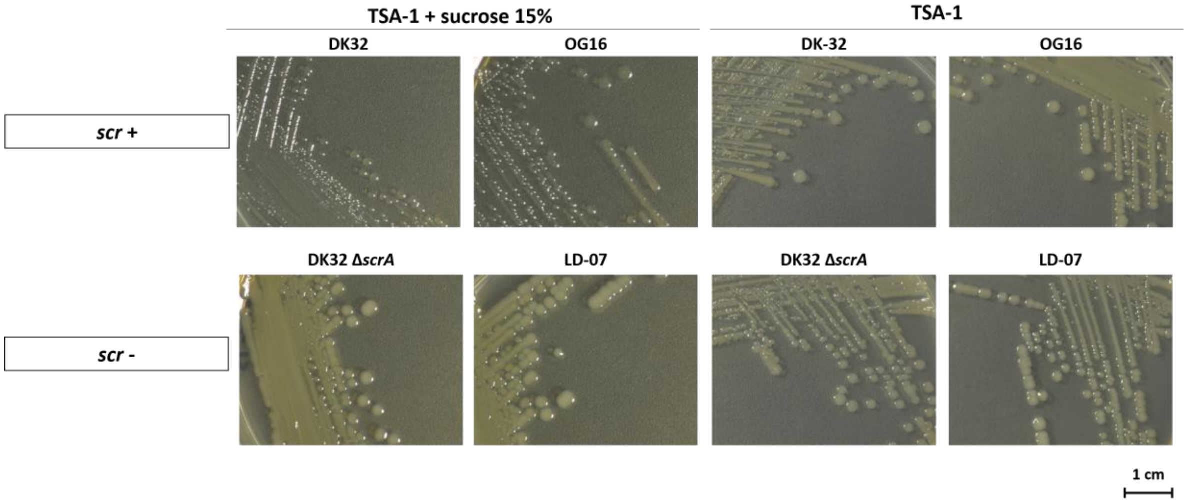

Figure S1: Colony morphologies of Scr⁺ and Scr⁻ *Pdd* strains grown in either plain TSA-1 or in TSA-1 supplemented with 15% sucrose. Strains harboring the *scr* gene cluster form flat and translucent colonies in presence of high concentrations of sucrose, while strains lacking a functional *scr* cluster form convex and whitish colonies. The colony phenotypes of Scr⁺ and Scr⁻ strains are indistinguishable on TSA-1 without added sucrose.
